# Electron Transport Properties of Graphene/WS_2_ Van Der Waals Heterojunctions

**DOI:** 10.3390/molecules28196866

**Published:** 2023-09-29

**Authors:** Junnan Guo, Xinyue Dai, Lishu Zhang, Hui Li

**Affiliations:** 1 Key Laboratory for Liquid-Solid Structural Evolution and Processing of Materials, Ministry of Education, Shandong University, Jinan 250061, China; guojunnan113005@hotmail.com; 2 Materdicine Lab, School of Life Sciences, Shanghai University, Shanghai 200444, China; dxy1120@shu.edu.cn; 3 Peter Grünberg Institut (PGI-1) and Institute for Advanced Simulation (IAS-1), Forschungszentrum Jülich, Jülich 52428, Germany; lis.zhang@fz-juelich.de

**Keywords:** graphene/WS_2_ heterojunctions, electronic transport, first-principles calculation

## Abstract

Van der Waals heterojunctions of two-dimensional atomic crystals are widely used to build functional devices due to their excellent optoelectronic properties, which are attracting more and more attention, and various methods have been developed to study their structure and properties. Here, density functional theory combined with the nonequilibrium Green’s function technique has been used to calculate the transport properties of graphene/WS_2_ heterojunctions. It is observed that the formation of heterojunctions does not lead to the opening of the Dirac point of graphene. Instead, the respective band structures of both graphene and WS_2_ are preserved. Therefore, the heterojunction follows a unique Ohm’s law at low bias voltages, despite the presence of a certain rotation angle between the two surfaces within the heterojunction. The transmission spectra, the density of states, and the transmission eigenstate are used to investigate the origin and mechanism of unique linear I–V characteristics. This study provides a theoretical framework for designing mixed-dimensional heterojunction nanoelectronic devices.

## 1. Introduction

Two-dimensional (2D) layered materials have always been a cutting-edge field in condensed matter physics and materials research [1]. Various 2D layers can be combined using van der Waals (vdW) forces to build heterostructures with diverse functionalities [2]. These heterostructures exhibit a range of excellent properties and are applied to optoelectronic devices [3], providing unprecedented opportunities for the development of advanced nanoelectronics devices [4].

As one of the atomically thin 2D materials, graphene [5] has attracted worldwide attention due to its excellent optical [6], electrical [7], and mechanical properties [8], and it is expected to be used to build a new generation of miniaturized and intelligent electronic devices [9]. However, the absence of a band gap has limited the application of graphene, particularly in the semiconductor industry [10]. Significant efforts have been devoted to addressing this issue in the gap-opening of graphene, like functionalization [11], doping [12], and the construction of heterostructures [13]. Recently, many graphene-based vdW heterostructures have been investigated theoretically and experimentally [14]. For instance, Lan et al. transferred graphene grown on a copper foil to a sapphire substrate with Bi_2_Te_3_ crystals via low-pressure chemical vapor deposition (CVD). The crystallized Bi_2_Te_3_ was synthesized directly using spin-coated coring (SCCA). This procedure avoided any degradation of the nanoplates and significantly improved the quality of the heterojunction sample [15]. Hu et al. utilized a polymethyl methacrylate (PMMA)/polydimethylsiloxane (PDMS) blend to transfer metal-catalyzed CVD-fabricated graphene/SiNWS heterojunctions onto stretchable polytetrafluoroethylene (PTFE) substrates. The high preparation efficiency and outstanding quality were extremely encouraging for daily industrial production and life [16]. Ren et al. developed a novel flexible self-powered photodetector that transfers electrons through a solid electrolyte. The developed flexible WS_2_/graphene photodetector displayed a quick photo response time and high photosensitivity [17]. Liu et al. fabricated Bi_2_Se_3_/graphene heterojunctions using molecular beam epitaxy and observed a spiral growth mechanism during the growth process [18]. By vertically stacking single-layer MoS_2_/h-BN/graphene, Lee’s team created random access memory with tunneling. It had excellent stretchability, long retention times, and highly dependable memory performance [19]. Additionally, Liu et al. investigated different conceivable atomic configurations of phosphorene/graphene in-plane heterojunctions and their effects on interfacial heat conductivity by using density functional theory calculations and molecular dynamics simulations [20]. Gao et al. simulated the heat transfer properties of graphene/MoS_2_ heterojunctions using nonequilibrium molecular dynamics simulations and found that the degree of lattice matching of graphene and MoS_2_ had an effect on phonon thermal transport [21]. However, the majority of these studies on graphene heterojunctions primarily focused on their electronic structures [22], preparation methods [23], and applications [24]. Little research has been conducted on their electron transport properties and intrinsic mechanisms. 

In this context, constructing new graphene heterojunctions and studying their electron transport properties are essential if one wants to realize the practical application of graphene heterojunctions in nanoelectronic devices. With excellent electron mobility and a large direct band gap, monolayer WS_2_ has a lot of potential uses in nanodevices [25]. In particular, in recent years, there have been significant breakthroughs in its synthesis and applications. For example, Prof. Feng’s group produced monolayer triangular WS_2_ single crystal wafers with excellent uniformity, large size, and high quality by controlling the nucleation density by changing the time of the introduction of the sulfur precursor and the distance between the tungsten source and the growth substrate [26]. Furthermore, some researchers have used chemical doping to significantly improve the optoelectronic performance of WS_2_ field-effect transistors [27]. Inspired by these advancements, we selected monolayer WS_2_ to create a series of graphene/WS_2_ heterojunction models and design nanoelectronic devices. We systematically investigated their electronic structures and transport properties using first-principles methods based on the density functional theory (DFT) and nonequilibrium Green’s function (NEGF) [28].

## 2. Results and Discussions

The hexagonal unit cell of WS_2_ was the same as that of graphene. For graphene and WS_2_, the optimized lattice parameters were 2.45 Å and 3.15 Å, respectively. The unit cell parameters we calculated closely matched experimental results [29,30].

To construct the graphene/WS_2_ heterojunctions, we used a 3 × 3 × 1 supercell of WS_2_ and a 4 × 4 × 1 supercell of graphene with 68 total atom numbers, and a 4 × 4 × 1 supercell of WS_2_ and a 5 × 5 × 1 supercell of graphene with 109 total atom numbers. In this orientation, both components maintained their original hexagonal lattices without surface rotation and exhibited slight lattice mismatches of 3.1% and 2.4%, respectively. The interlayer spacings of the equilibrium geometries of these two heterojunctions were 3.41 Å and 3.46 Å, respectively, which are typical distances in graphene-based vdW heterostructures with weak interactions.

**Figure 1:**
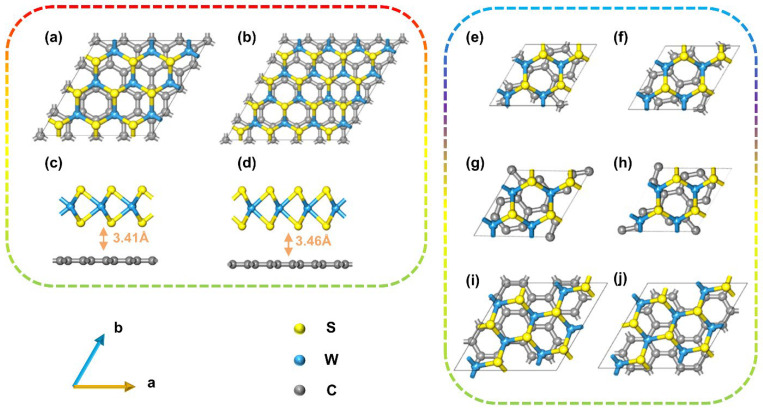
Top views of (**a**) Gr/WS_2_-1, (**b**) Gr/WS_2_-2, (**e**) Gr/WS_2_-3, (**f**) Gr/WS_2_-4, (**g**) Gr/WS_2_-5, (**h**) Gr/WS_2_-6, (**i**) Gr/WS_2_-7, and (**j**) Gr/WS_2_-8 ball-and-stick models. Side views of (**c**) Gr/WS_2_-1 and (**d**) Gr/WS_2_-2.

However, the devices built from the above two heterojunctions contained 366 and 603 atoms, separately. Due to the limitations of quantum-mechanics-based calculations used in this study, we continued to construct a series of heterojunctions with specific rotation angles between each surface to reduce the models’ sizes. In these heterojunctions, the interatomic distances were consistently around 3.4 Å, indicating weak vdW interactions. At the same time, we could also analyze the electron transport properties of devices that had different rotation angles. The equilibrium geometries of heterojunctions and their related parameters are shown in Figure 1 and Table 1.

In order to prove the thermodynamic stability of these heterojunctions, the binding energies of the graphene/WS_2_ vdW heterojunctions were calculated to assess the system stability, as follows:

$$E_{b}=E\left(heterojunction\right)-E\left(graphene\right)-E(WS_{2})$$

where *E*(heterojunction), *E*(graphene), and *E*(WS_2_) represent the total energy of the heterojunctions, graphene layers, and WS_2_ layers, respectively. The calculated binding energies are presented in Table 2. The negative binding energies in the table indicate the stability of these systems. Upon comparison, we observed that the most stable heterojunction was Gr/WS_2_-1. Another regularity we found was that smaller heterojunctions were more stable when the two layers were not rotated. However, when there exist rotation angles between the two layers, the stability of the heterojunctions decreased and the larger heterojunctions were more stable.

**Figure 2:**
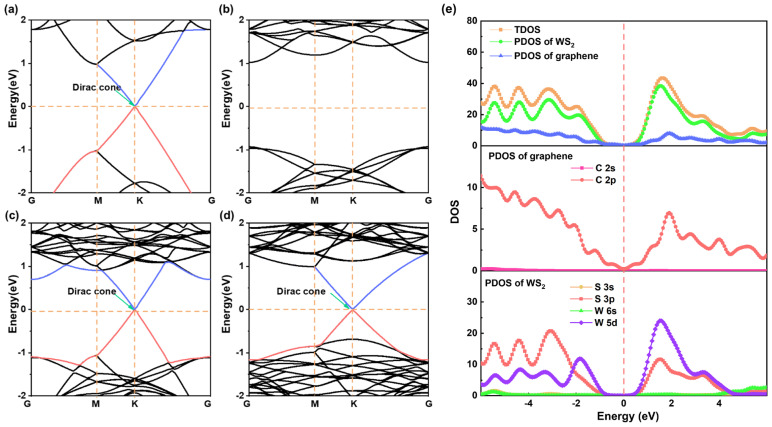
Band structures of the stand-alone (**a**) graphene and (**b**) WS_2_; (**c**,**d**) are band structures of Gr/WS_2_-1 and Gr/WS_2_-2. The red and blue lines represent the top of the valence band and the bottom of the conduction band. (**e**) The PDOS and DOS of the graphene and WS_2_ components in the vdW Gr/WS_2_-1.

We initially investigated the electronic properties of Gr/WS_2_-1 and Gr/WS_2_-2 to determine whether they can be transported as electronic devices. As plotted in Figure 2a, graphene exhibits metallic properties with a zero bandgap semiconductor, where the top valence band and bottom conduction band intersect at the K point. In contrast, WS_2_ is a semiconductor with a direct band gap of 1.95 eV, as shown in Figure 2b. It is worth noting that our calculations closely aligned with other theoretical predictions and were slightly lower than experimental values [31]. This discrepancy can be attributed to the inherent limitations of the GGA-PBE method, which tends to overestimate lattice constants and underestimate band gaps. Hybrid functionals, such as meta-GGA, HSE06, etc., are known to provide more accurate bandgap calculations [32]. However, the WS_2_ bandgap calculated by meta-GGA was 2.13 eV, which was only slightly higher than the PBE value (1.95 eV). Thus, we believe that the GGA-PBE approach was accurate enough for our calculation and did not significantly impact other aspects of the analysis, such as energy band structure and electron transport.

Figure 2c and d display the band structures of Gr/WS_2_-1 and Gr/WS_2_-2, which are simple superpositions of graphene and WS_2_ and preserve their electronic systems. Notably, the valence band’s top and the conduction band’s bottom still intersected at the K point in the Brillouin zone, indicating that the Dirac point still exists in the heterojunction. Gr/WS_2_-1 behaved as an N-type semiconductor, with the E_c_ and E_v_ of WS_2_ shifting downwards. Additionally, the Fermi energy level turned from near the top of the valence band to near the bottom of the conduction band. Conversely, Gr/WS_2_-2 exhibited P-type semiconductor properties, with the Fermi energy level still close to the top of the valence band, but the conduction band bottom and valence band top shifted from the original G to the K point. This indicates that factors such as layer spacing, the degree of mismatch, and lattice parameters within the heterojunction influence its electronic energy band.

Next, we calculated the density of states (DOS) and the projected calculation density of states (PDOS). Due to the similarity in the calculation results, we present the results for Gr/WS_2_-1 as an example. According to Figure 2e, near the Fermi level, the 2p orbital of the carbon atom in graphene plays a vital role in the density of states. The 5d orbital of the W atom also makes a contribution. Contributions from other valence electron orbitals can be disregarded. The absence of resonance peaks indicates that there was no bonding between WS_2_ and C. Instead, weak van der Waals forces maintained the interlayer stability between the heterojunctions, corresponding to optimized interlayer spacing of around 3.4 Å. This weak hybridization between the graphene and WS_2_ is another indication of why the graphene’s Dirac points are still present in the heterojunctions.

**Figure 3:**
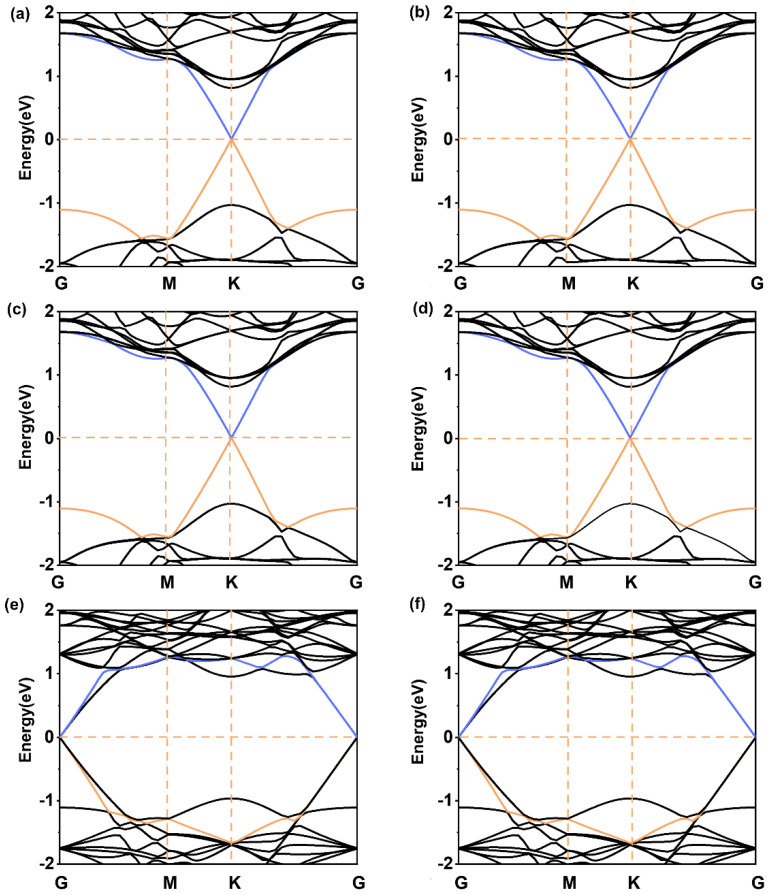
Band structures of (**a**) Gr/WS_2_-3, (**b**) Gr/WS_2_-4, (**c**) Gr/WS_2_-5, (**d**) Gr/WS_2_-6, (**e**) Gr/WS_2_-7, and (**f**) Gr/WS_2_-8. The orange and blue lines represent the top of the valence band and the bottom of the conduction band.

As depicted in Figure 3, when there is a certain rotation angle between the two surfaces, no matter the change in the lattice constants or the rotation angle of graphene or WS_2_, its effect on the energy band is little. But when the lattice parameter of heterojunctions is increased to around 8 Å, the Dirac cone of graphene shifts from K to G point due to the inequivalent K and K’ points being folded and coupled into the same G-point (Figure 3e,f). However, the Dirac cone does not open. We predicted that these six heterojunctions had comparable electronic transport properties. Consequently, nanoelectronic devices could be built using heterostructures with rotation angles to reduce device size while maintaining their high transport properties.

**Figure 4:**
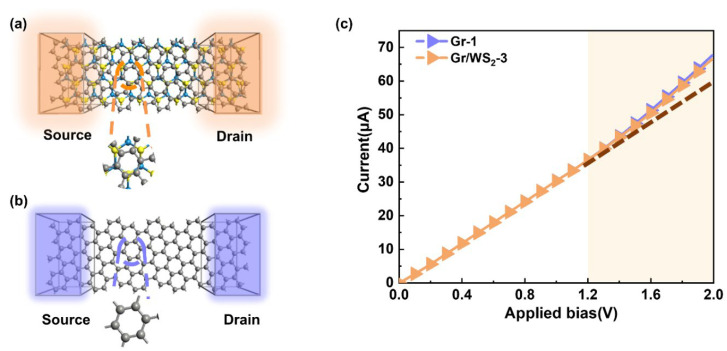
The device configuration with (**a**) Gr/WS_2_-3 and (**b**) Gr-1. (**c**) I–V characteristics of devices.

With the Gr/WS_2_-3 and Gr-1 (composed of graphene, with the same lattice parameter and rotation angle as Gr/WS_2_-3), we built two devices, as depicted in Figure 4. As seen in the enlarged area, the rotation angle between graphene and WS_2_ was still maintained. The poles of the device formed by themselves, the current transport direction was along the Z-axis, and the surface was perpendicular to the X-axis.

The I–V characteristics of the devices in a bias zone [0.0 V, 2.0 V] were calculated to explore the transport characteristics of these two devices, and the findings are shown in Figure 4. We can see from the current-voltage (I–V) characteristic curves (Figure 4c) that the heterojunction had comparable transport properties to graphene, unlike some typical heterojunction semiconductor devices. Interestingly, the Ohmic behavior of linear I–V curves was found in the 0–1.2 V bias voltage. After 1.2 V, the slope of the I–V curve gradually increased, leading to nonlinear transport properties. This was caused by a certain degree of rotation in the graphene and heterojunction, while the transport direction was primarily along the armchair direction of the graphene. Simultaneously, it became evident that the transport properties of both devices changed gradually as the voltage increased, signifying a weakened coupling between WS_2_ and graphene. A nonlinear relationship only began to emerge at high bias voltages. Compared to other graphene-based heterojunctions, the transport current of graphene/WS_2_ was nearly one order of magnitude higher than that of graphene/MoS_2_ in-plane heterojunctions [33,34], graphene/BN heterojunctions [35], and so on. In addition, when compared to other WS_2_-based heterojunctions, such heterojunctions could behave up to two orders of magnitude higher than that of WS_2_/WSe_2_ heterojunctions [36], with greater performance than that of MoS_2_/WS_2_ heterojunctions [37]. Thus, we can conclude that such heterojunctions can greatly enhance the transport current and decrease the contact resistance, which will be very important for achieving superior optoelectronic devices such as vertical field-effect transistors (FETs). Our calculations can reveal why graphene/WS_2_ heterojunctions are widely used to build FETs and have superior behavioral properties [38,39,40,41]. In addition, the heterojunction used in our calculations not only maintained the perfect transport properties but also largely reduced the size of the electronic devices, which is very important in the post-Moore era.

Although the differences in transport properties between these two devices were slight, the transport mechanism exhibited different phenomena due to the weak vdW forces between the WS_2_ and graphene. The most understandable depiction of the behavior of electron transport was the transmission spectrum *T*(*E*), and the transmission coefficient of each energy point was determined by diagonalizing the transmission matrix from the eigenvalues of electron transmission. Therefore, we calculated the transmission spectra of the above devices to further study their transport properties. 

**Figure 5:**
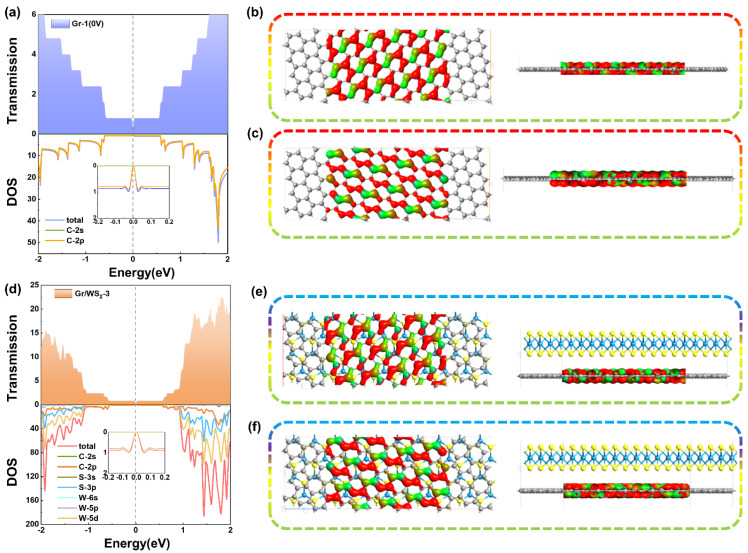
Transmission spectrum and DOS of (**a**) Gr-1 device and (**d**) Gr/WS_2_-3 device at a free applied bias (0.0 V). Transmission eigenstate of Gr-1 (**b**,**c**) and Gr/WS_2_-3 (**e**,**f**) around Fermi level with an isovalue of 0.21.

Generally speaking, the magnitude of the transmission coefficient near the Fermi level represents the transport capability of the device, especially at the Fermi level. The larger the transmission coefficient at the Fermi level, the stronger the transport capability. As shown in Figure 5a,d, these two devices exhibited metallic properties, corresponding to the current–voltage curves. The electron transmission spectra of graphene devices and Gr/WS_2_-3 devices displayed quantum steps between −1 eV and 1 eV, resembling the ideal one-dimensional nanowires. And the electron transmission probability at the Fermi energy level was almost zero, which shows a band gap feature between the conduction and valence bands, corresponding to a Dirac cone in the energy band structure. Although the system had almost no electrons passing through at this energy, at higher energies electrons could easily tunnel through the potential barrier, increasing their mobility and the step transmission coefficient, which indicates that there were several electron transmission channels in these devices. After the formation of the heterojunction, many spikes appeared away from the Fermi energy level, showing that the coupling between the graphene and WS_2_ was weak. The band gap of graphene was not open, although it tends to be open, which does not have a significant influence on its transport properties. 

To further shed light on the inherent mechanisms of these two devices, we discuss DOS around the Fermi level for these devices. Figure 5a,d illustrate that the DOS of the two devices were zero at the Fermi energy level, corresponding to their electron transmission spectrum. Before the construction of the heterojunction, the contribution of DOS near the Fermi energy level originated mainly from the 2p orbitals of the graphene carbon atoms. After the formation of the heterojunction, the contribution was mainly from the 2p orbital of the graphene carbon atom and the 5d of the W atom. We can see clearly that several peaks exceeded 100 in the Gr/WS_2_-3, more than twice that of the Gr-1. The highest peaks in the valence band region were observed at −1.92 eV, while those in the conduction band region were found at 1.44 eV. These peaks serve to protect fewer delocalized states near the Fermi level.

Here, the dominant transmission eigenstates near the Fermi energy level at equilibrium were calculated to explore the physical roots of their transport phenomena. The calculated results in Figure 5b,c,e, and f showed that the transmission of two devices around the Fermi level was provided by two major transport channels, both with transmission eigenvalues of nearly 1.000. The transmission eigenstates of both devices exhibited delocalization throughout the whole central region, resulting in significant transport capability near the Fermi energy level. We can see that the electronic states were evenly distributed in the diffusion region between the left and right electrodes, along the graphene armchair direction. This indicates that these states were all π-orbitals of the C atom of graphene, leading to their metallic characteristic. However, the contribution of WS_2_ in Gr/WS_2_-3 was almost negligible.

**Figure 6:**
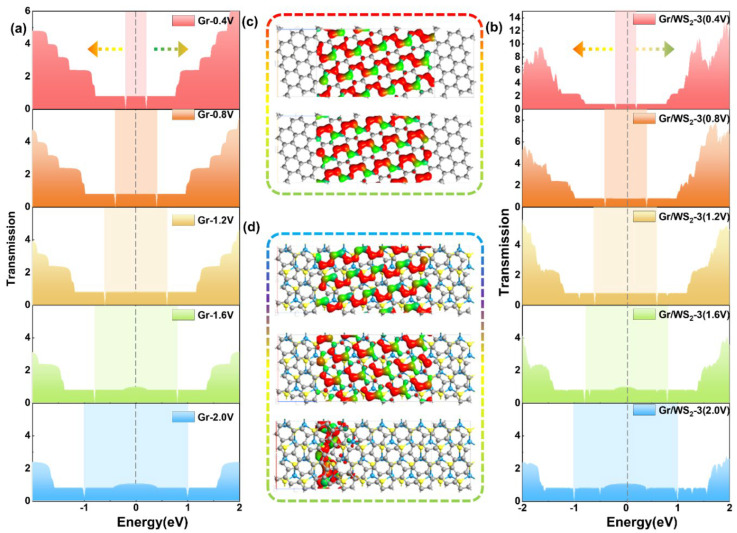
Transmission spectra of (**a**) Gr-1 device and (**b**) Gr/WS_2_-3 device at nonzero bias voltage. Transmission eigenstates of Gr-1 (**c**) and Gr/WS_2_-3 (**d**) at 2.0 V with an isovalue of 0.21. The colored arrows represent the movement of the steps in the conduction and valence band regions.

It is well known that the study of transmission spectra at non-zero bias voltages can provide useful information for the study of I–V characteristics. This is because the current is defined by the integrated area of the transmission curve within the bias window, as shown by the Landauer–Buttiker formula. As a result, we calculated the transmission spectra of the Gr-1 and Gr/WS_2_-3 devices under 0.4, 0.8, 1.2, 1.6, and 2.0 to further reveal their transport phenomena (Figure 6a,b). The bias window’s perimeter is represented by the colored parts. The effective integral area of the transmission curve within the bias window grew with increased bias, producing a linear I–V characteristic, as we can see from both devices. However, when the bias window increased to 1.2 V, the step transmission spectrum started to change shape and expand in an arc, so the I–V curve began to show non-linear features, and the slope subsequently increased. It is evident from the transmission spectrum that quantum steps are always present within the bias window at different bias voltages and that the steps shift as the bias window expands. The movement tendency of the steps in the conduction and valence band regions was indicated by the arrows, respectively. The number of wave valleys within the bias window in the Gr/WS_2_-3 devices progressively increased. Spikes far from the Fermi energy level moved in the opposite direction and were unable to move inside the bias window, so the contribution of these spikes to the transport properties was almost negligible. Interestingly, the lowest transmission probability was always located at the boundary of the bias window, and as the bias increased from 0 V to 2 V, the gap shifted to the boundary of the bias window.

It is worth noting that the transmission eigenstates of electrons may change under external bias after forming the heterojunction. Therefore, the electronic transmission eigenstates of Gr-1 and Gr/WS_2_-3 devices were calculated at different bias voltages. Before 2.0 V, the transmission eigenstates of both devices were mainly contributed by the two transmission channels of graphene. However, when the bias voltage increased to 2.0 V, the transmission channels at the Fermi level of Gr/WS_2_-3 changed from two to multiple channels, as shown in Figure 6c,d. WS_2_ started to participate in the transport, but its electronic state was localized at the left electrode and the transmission eigenvalue was so small that it can still be disregarded. 

## 3. Computational Method

DFT implemented in the Atomistix ToolKit (ATK) package was used to optimize the geometry structures and calculate the electronic structures of graphene/WS_2_ heterostructures. The exchange–correlation function is a generalized gradient approximation (GGA) [42] of Perdew–Burke–Ernzerhof (PBE) [43]. The selected valence electron configurations in our calculation were W 5d^4^ 6s^2^, S 3s^2^ 3p^4^, C 2s^2^ 2p^2^. In order to meet the computational precision, the linear combination of atomic orbitals (LCAO) basis was selected for all atoms. Double-ζplus polarization (DZP) basis sets were adopted for the local atomic numerical orbitals, and norm-conserving pseudo-potentials were employed. The Monkhorst–Pack k-points of 5 × 5 × 1 were used to sample the Brillouin zone [44]. The cut-off energy for the density mesh and the electron temperature were set to 75 Ha and 300 K, accordingly.

The device’s performances were studied with the DFT coupled with the NEGF method, using the ATK package. A 15 Å vacuum layer along the X-direction was used to avoid interactions between periodic images that were nearest neighbors. For self-consistent calculation, the k-points of 5 × 5 × 100 were used for device models. The other parameters of the DFT calculation remained unchanged, and the energy convergence criterion was set to 10^−4^ eV. Before analyses, the devices were fully optimized by the quasi-Newton approach until all residual stresses on each atom were less than 0.05 eV. The devices’ electronic properties were investigated by computing their currents, the density of states, and the transmission spectra, and the current *I* through the device was calculated using the Landauer–Buttiker equation [45]:

$$I=\frac{2e}{h}\int_{-\infty }^{+\infty }dE(T\left(E,V\right){(f}_{1}\left(E\right)-f_{2}(E)))$$

The quantity $T\left(E\right)$ is the transmission function, which expresses the likelihood that electrons will go through the device from source to drain; $f_{1,2}(E)$ denote the Fermi functions of the source and drain electrodes; and e and h are the electron charge and Planck’s constant, respectively.

## 4. Conclusions

In this work, we systematically studied the electronic transport properties and intrinsic mechanisms of graphene/WS_2_ heterojunctions using first-principal calculations. Unique linear I–V characteristics were found among the devices. Even though there was an angle between the two surfaces, the heterojunction continued to exhibit this intriguing Ohm’s law behavior. The transmission spectra, the density of states, and the transmission eigenstate were calculated to explain this phenomenon. After forming the heterojunctions, the quantum steps near the Fermi level approximated an ideal one-dimensional nanowire. The DOS shows that the vdW heterojunctions significantly increased the number of peaks and improved the maximum value of peaks, which protected less delocalized states near the Fermi level. The transmission eigenstates showed that the high transport properties came from the π orbitals of the C atoms in the graphene armchair direction. This study provides valuable insights into the transport properties of graphene heterojunctions and the potential fabrication of mixed-dimensional heterojunctions.

## Figures and Tables

**Table 1:** The related parameters of heterojunctions.

Heterojunction	Lattice Parameters of Graphene (Å)	Rotation Angle of Graphene (°)	Lattice Parameters of WS_2_ (Å)	Rotation Angle of WS_2_ (°)	Lattice Mismatch (%)
Gr/WS_2_-1	a = b = 9.8	0.0	a = b = 9.5	0.0	3.1
Gr/WS_2_-2	a = b = 12.3	0.0	a = b = 12.6	0.0	2.4
Gr/WS_2_-3	a = b = 6.5	21.8	a = b = 6.3	60.0	3.1
Gr/WS_2_-4	a = b = 6.5	141.8	a = b = 6.3	60.0	3.1
Gr/WS_2_-5	a = b = 6.5	21.8	a = b = 6.3	180.0	3.1
Gr/WS_2_-6	a = b = 6.5	141.8	a = b = 6.3	180.0	3.1
Gr/WS_2_-7	a = b = 8.5	0.0	a = b = 8.3	21.8	2.1
Gr/WS_2_-8	a = b = 8.5	120.0	a = b = 8.3	21.8	2.1

**Table 2:** The binding energy of heterojunctions.

Heterojunction	Energy of Graphene (eV)	Energy of WS_2_ (eV)	Energy of Heterojunction (eV)	Binding Energy (eV)
Gr/WS_2_-1	−5038.3	−10,206.0	−15,246.9	−2.6
Gr/WS_2_-2	−7874.7	−18,145.4	−26,022.2	−2.1
Gr/WS_2_-3	−2204.4	−4563.7	−6741.6	−0.6
Gr/WS_2_-4	−2204.4	−4563.7	−6741.6	−0.6
Gr/WS_2_-5	−2204.4	−4563.7	−6741.6	−0.6
Gr/WS_2_-6	−2204.4	−4563.7	−6741.6	−0.6
Gr/WS_2_-7	−3779.3	−7939.2	−11,719.4	−0.9
Gr/WS_2_-8	−3379.3	−7939.2	−11,719.4	−0.9

## Data Availability

Data will be made available on request.
